# The Effect of a Single 30-Min Long Term Evolution Mobile Phone-Like Exposure on Thermal Pain Threshold of Young Healthy Volunteers

**DOI:** 10.3390/ijerph15091849

**Published:** 2018-08-27

**Authors:** Zsuzsanna Vecsei, György Thuróczy, István Hernádi

**Affiliations:** 1 Department of Non-ionizing Radiation, National Public Health Institute, H-1221 Budapest, Hungary; vecsei@hp.osski.hu (Z.V.); thuroczy@hp.osski.hu (G.T.); 2 Department of Experimental Zoology and Neurobiology, Faculty of Sciences, University of Pécs, H-7624 Pécs, Hungary; 3 Szentágothai Research Center, and Center for Neuroscience, University of Pécs, H-7624 Pécs, Hungary; 4 Institute of Physiology, Medical School, University of Pécs, H-7624 Pécs, Hungary

**Keywords:** long term evolution, LTE, 4G, mobile phone, nociception, pain, thermal pain threshold

## Abstract

Although the majority of mobile phone (MP) users do not attribute adverse effects on health or well-being to MP-emitted radiofrequency (RF) electromagnetic fields (EMFs), the exponential increase in the number of RF devices necessitates continuing research aimed at the objective investigation of such concerns. Here we investigated the effects of acute exposure from Long Term Evolution (LTE) MP EMFs on thermal pain threshold in healthy young adults. We use a protocol that was validated in a previous study in a capsaicin-induced hyperalgesia model and was also successfully used to show that exposure from an RF source mimicking a Universal Mobile Telecommunications System (UMTS) MP led to mildly stronger desensitization to repeated noxious thermal stimulation relative to the sham condition. Using the same experimental design, we did not find any effects of LTE exposure on thermal pain threshold. The present results, contrary to previous evidence obtained with the UMTS modulation, are likely to originate from placebo/nocebo effects and are unrelated to the brief acute LTE EMF exposure itself. The fact that this is dissimilar to our previous results on UMTS exposure implies that RF modulations might differentially affect pain perception and points to the necessity of further research on the topic.

## 1. Introduction

The continuing development of telecommunication technology throughout the twentieth century necessitated the increasing density of devices emitting electromagnetic fields (EMF). Especially with the advent of new generation mobile phone (MP) technologies, wearables and the forthcoming “internet of things,” EMF generating devices are becoming more abundant, they are utilized more frequently and generally closer to the body of the user. Therefore, investigating the potential health risks and adverse effects of these devices has never been more important.

Most people do not experience any symptoms due to regular MP use. However, a small portion of the population (suffering from the condition called Idiopathic Environmental Intolerance Attributed to Electromagnetic Fields, IEI-EMF) connects non-specific symptoms to electromagnetic field (EMF) exposure [1], especially from mobile phones [2]. The symptoms include dizziness and sleep disturbances but the most prevalent and important of them is pain, mostly in the form of headaches. Attempting to determine whether the experience of these patients have any objective basis or is solely due to psychological factors is one important approach in the search for potential EMF effects on human health and well-being [3,4].

Research using model organisms such as mice, snails and rats have yielded mixed results, ranging from hyperalgesia through null effects to analgesia depending on the EMF applied and the operationalization of “pain” in the experimental paradigm applied. For example, repeated magnetic field (MF) exposure accompanied by extremely low frequency (ELF) EMF exerted analgesic effects in mice and in land snails [5,6]. In contrast, acute exposure (30 min) of specific ELF MFs demonstrated consistent inhibitory effect on opioid-induced analgesia [7,8,9,10]. In peripubertal rats, the chronic (45 days) and intermittent (2 h/day) exposure to amplitude modulated RF EMF facilitated the emotional component of phasic pain, while late responses to tonic pain stimuli were decreased [11].

Besides the variability in these results, it is important to note that human pain sensation is known to be a complex phenomenon depending on a the interplay of sensory and cognitive-affective factors [12,13], thus well designed human experimental studies are of prime importance to inform us about the relevance of animal neurophysiological studies with respect to the pain-related aspects of human EMF sensitivity. For example, both in humans and animal models, EMFs with sufficiently high energy are known to increase the temperature of the exposed tissue, which results in thermal noxious stimulation similar to that caused by other types of heating effects [14]. However, everyday human EMF exposure occurs very far from this range since appliances in regular human use are restricted in power exactly to avoid possible tissue damage or painful heating effects [15].

Epidemiological work has also been conducted to probe the possible relations between mobile phone use and the symptoms sometimes attributed to it [2,16,17,18,19]. These studies have the advantage of the large, representative sample and high ecological validity but the information they can provide on the objective reality and potential mechanisms behind pain-related EMF effects is very limited. The main problem is that these studies generally cannot differentiate between the potential effects from radiofrequency (RF) EMF exposure and psychological aspects of mobile phone use and they generally do not provide information on the exact personal exposimetry of EMF. Under well-controlled experimental conditions symptoms are not clearly associated with real RF exposure [20]. These human provocation studies provided evidence that it is the so called ‘nocebo’ effect (that is the influence of negative expectations on the effect of the treatment) that determines the symptoms, not the exposure itself [21,22,23].

Methods to measure painful experiences attributed to EMFs in previous studies mostly involved questionnaires and visual analogue scales (VAS) or analyzing subjective verbal reports. These methods measure the experience of pain integrating across the domains of basic sensory nociception up to the highest levels of cognitive and affective appraisal. This can be considered an advantage but it is also important how these different stages might contribute to potentially altered perception of pain due to EMF exposure. Well before the stage when the degree of pain is distilled to a number on a scale or into the form of a verbal statement, one of the earliest behavioral markers of pain is the abrupt withdrawal of the affected limb from the noxious stimulus [24]. In our previous study [25], we have designed a device and methodology to assess thermal pain threshold (TPT) by measuring the level of thermal stimulation that induces limb withdrawal. The methodology was validated using the capsaicin-induced hyperalgesia model. It was successfully used to examine the effects of UMTS (Universal Mobile Telecommunication System) RF emitted by third generation (3G) MPs on the pain threshold in healthy young adults in a provocation study with a double-blind, placebo-controlled crossover design [25]. The purpose of the present study was to examine similar possible effects of LTE (Long Term Evolution) RF emitted by the fourth generation (4G) MPs on the temperature pain threshold, using an experimental design fully consistent with our previous UMTS study.

## 2. Materials and Methods

### 2.1. Participants

Participants were full-time students of the University of Pécs. Eighteen healthy adults (12 females, 6 males, age range: 19 to 26 years, mean age ± SEM: 21 ± 2 years) participated in the experiment. They reported painless health without any medication. All participants were right-handed and generally used their right hand for phone calls by self-report. All participants signed a written informed consent after the experiment was fully explained. They were compensated by practical course credit for the time spent in the experiment. They were informed that they can withdraw from their intention to participate in the experiment at any time, without falling away from the offered compensation. The study was conducted in accordance with the declaration of Helsinki and the protocol was approved by the Regional and Institutional Research Ethics Committee of the University of Pécs (file number: 5349).

### 2.2. Heat Stimulation Device

The heat stimulation device (LRG. Kft., Győr, Hungary) applied here was a newly re-designed version of an earlier device used and validated in our previous study [25] on thermal pain threshold. The heat stimulating device used in this study consisted of a computer-controlled miniature piezoelectric heating pad (heating surface approx. 5 × 5 mm^2^), a 3D motion detector (accelerometer) and a precise digital temperature sensor (with one decimal digit (°C) accuracy). The sensor of the device was attached stabile but easy removable to the inner surface of the tip of the index finger. The heat stimulus was the increasing surface temperature of the heating pad with adjustable temperature range and increase ramp rate. In the current study—like in the previous one—the temperature was increased with a rate of 5 °C/s from 35 °C up to maximum 55 °C. When the stimulated finger quickly moved (over 150 mg acceleration value), the heating phase was immediately interrupted and the device was actively cooled down to the initial temperature within 2 s. At the moment of the trial termination the heating pad temperature was logged on a PC microcomputer at 100 Hz sampling rate. In order to avoid accidents, when the heating pad temperature reached 55 °C, the heating phase was interrupted even if the participants did not respond to the stimulation. In such case, 55 °C was considered as the temperature pain threshold.

### 2.3. LTE Exposure System

**Figure 1:**
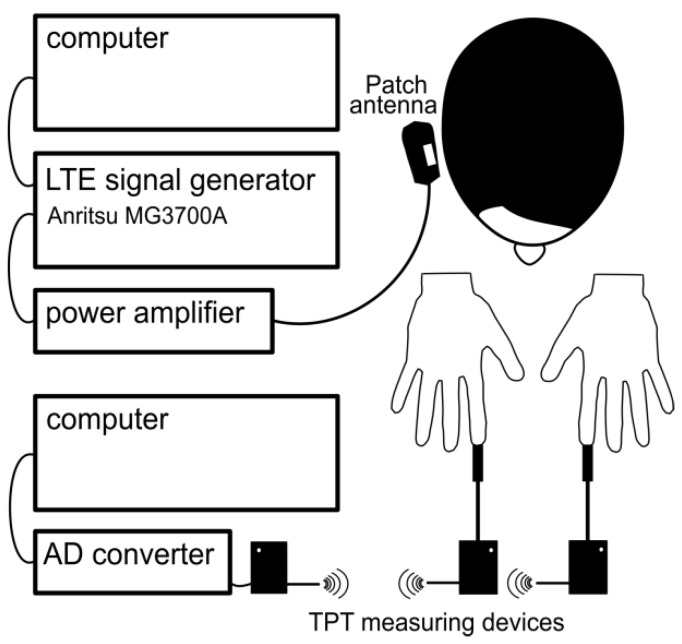
Experimental arrangement scheme. The LTE radiofrequency exposure system (top left) and the thermal pain threshold measuring device (bottom right).

The LTE RF exposure system was set up a programmable signal generator, power amplifier, the antenna holding fixture a patch antenna (see Figure 1). The LTE “signal cocktail” was generated by an Anritsu MG3700A (Anritsu Co., Kanagawa, Japan) programmable signal generator. The signal generator was remotely controlled by a computer interface. The generator and the screen were not visible to the investigator nor the participants, either enabling or disabling signal output (corresponding to Real or Sham exposure, respectively), thus enforcing double-blind experimental conditions. For this experiment 1750 MHz (corresponding to one of the operating frequencies of LTE systems in Europe) was used as the carrier frequency with 20 MHz of bandwidth (the allowed maximum of LTE signal). The signal generator was connected to the RF power amplifier BPAM14 (Bonn Hungary Electronics Ltd., Budapest, Hungary). The maximum peak SAR was to 1.8 W/kg and the ear-patch antenna distance to 7 mm. These values determine the input power to be 2.72 W (34.35 dBm). 

#### 2.3.1. LTE Signal

The LTE “signal cocktail” was generated by Anritsu MG3700A (Anritsu Co., Kanagawa, Japan) programmable signal generator. This device is able to generate an arbitrary baseband waveform and mix it on a RF carrier of arbitrary frequency and power.

Unlike the GSM (2G) and WCDMA (3G) standards, which are based on single-carrier modulation schemes, the LTE physical layer is based on Orthogonal Frequency Division Multiplexing (OFDM) technology. The fundamental principle of OFDM is to use a large number of narrowband, orthogonal subcarriers for data transmission instead of using a single, wideband carrier. This modulation and signal processing in this multicarrier approach allows not only for better spectral efficiency but also reduces the impact of multipath reflections on the receiver’s ability to demodulate the signal. The narrowband carrier signals have different phase and modulation, therefore the average envelope of all modulations and carriers within the 20 MHz signal bandwidth resemble a CW signal with very low modulation depth. The 20 MHz bandwidth (this is the maximum allowed by the LTE standard) is also specific to the LTE signal, since 3G and 2G have bandwidths of 5 MHz and 200 kHz, respectively.

The specific LTE signal used in the experiment was first created on a PC using the IQproducer V.7 software (Anritsu Corporation, Kanagawa, Japan), then the signal sequence was uploaded to the RF signal generator. During the experiment, the signal generator was remote controlled via TCP/IP by our custom-developed driver software. We selected the 16 QAM modulation waveform where all 100 resource blocks possible in this configuration were active. It should be noted that such signals rarely occur in practice however it approximates well the "worst case" therefore may cause the highest possible exposition. The signal generator was connected to RF power amplifier BPAM14 (Bonn Hungary Electronics, Budapest, Hungary) operating between 1700–2000 MHz, with a maximum output power of +41 dBm. Our previous measurements revealed that the SAR pattern generated by the patch antenna was localized close to the area of the antenna surface and was highly concentrated there [26].

#### 2.3.2. Patch Antenna

The exposure device was based on a single-sided round dual band patch antenna (Reinheimer Elektronik, Wettenberg, Germany; model no: M30EXO-0250-XX) with resonant surface and ground plane on the same side. The antenna was tuned with a waveguide resistor resonator and capacitors (SMD realization). This antenna arrangement provided sufficiently enhanced local exposure at the ear region. The 31-mm-diameter 0.5-mm thick patch antenna was encapsulated in a 40-mm-diameter 7-mm-thick transparent plastic capsule with Styrofoam thermal isolation. Reflection measurements demonstrated that the capsule did not have any significant effect on the antenna’s reflection and radiation parameters nor the effectiveness. The non-metallic side of the antenna was designated to be the application side faced to the ear. The covering case on this side was 1 mm thick. The antenna described here was developed earlier and was used in earlier investigations already published [26,27]. A plastic holding headset with spherical joints allowed precise, repeatable antenna positioning on the head of the subjects. The patch antenna was mounted in a position mimicking the normal use of an MP, the center of the patch antenna was near the exit of the ear canal, above the tragus, at a distance of 7 mm. The EEG cap compressed the pinna, allowing more precise positioning.

#### 2.3.3. Measurement of Specific Absorption Rate (SAR)

The measurements of absorbed radiofrequency power in the head, termed specific absorption rate (SAR, W/kg), were made in a standard human head SAM phantom (Specific Anthropomorphic Mannequin phantom, Antennessa, France) filled with standard brain tissue-equivalent liquid (Satimo, France) according to CENELEC standard EN 50361 (CENELEC, 2002). A small electric field probe (O6-EP64, Satimo, France) connected to a microvoltmeter (Keithley, OR, USA) was used for the measurement of the electric field strength within the liquid. The liquid calibration was based on an open-ended coaxial cable immersed in the phantom liquid connected to a vector network analyzer (Wiltron 360B, Morgan Hill, CA, USA). The electric field probe motion was realized by a servo-driven XYZ positioning system with a 3D step motor robot (Charlyrobot, Mecanumeric, Albi, France).

### 2.4. Experimental Design

**Figure 2:**
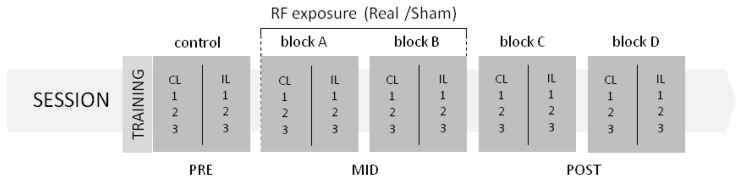
Timeline of the experimental design. In two sessions, each of the five consecutive blocks (control, block A, block B, block C, block D) consisted of six trials with three—three trials at the one (CL, contralateral) and at the other (IL, ipsilateral) hand. Thirty minutes after the control block (at the beginning of Block A), the half-hour exposure started: in the one session, the real (Real), and in the other the (Sham). In the last five minutes of the irradiation, the TPT data of Block B was performed. After exposure (Post) TPT’s were measured in two additional blocks (block C, block D). Half an hour passed between each block, with one minute inter stimulus intervals within one block.

A double-blind, counterbalanced experimental design was used to examine the effect of RF exposure on TPT, the same as of our previously published study [25] on TPT. The timeline of one session is shown in Figure 2. Two separate experimental sessions were conducted with at least a one-week interval between the sessions—Real and Sham exposure sessions. Both sessions were carried out at an identical time of the day in the morning (between 8 and 12 am) or in the afternoon (between 12 and 6 pm), balanced across participants to avoid possible interference with circadian regulation effects [28]. Participants were instructed to abstain from smoking, alcohol, caffeine and capsaicin-containing food consumption at least six h prior to the investigation and to moderate phone use before investigation (less than a total of 60 min on the day of the session). Each participant met the expected criteria—according to their declaration. After that experiment was fully explained (both in writing and verbally) and participants signed a written informed consent, they sat comfortably in an air-conditioned room.

The heat stimulator device was attached to the fingertips of the left and right index fingers. Participants were asked to quickly move their stimulated finger at the moment as their sense of warmness distinctly turns into pain. The movement of the finger terminated the trial and the temperature of the heating surface—at exactly the moment of movement—was registered as the TPT. The order of sessions (Real, Sham), the time of the day (am, pm) and the gender were counterbalanced across participants. Each session consisted of five blocks of trials. Participants performed two practice trials with each index finger before the first block. In each block, index fingers were stimulated six times (three trials per finger) in a random order with 60 s inter stimulus interval. The first block served as the control block. Thirty minutes after the control block, the right side of the participants’ head (mimicking normal MP use) was Real or Sham exposed. Thermal pain threshold was measured in two blocks during exposure (Mid): in the first block (Block A) at the beginning and in another block (Block B) at the end of the exposure phase. Participants performed two additional blocks at 30 min and at 60 min after the exposure phase (Post, Block C and Block D). Thus, each participant carried out a total of 60 trials in two separate experimental sessions: 2 sessions × (5 blocks × 2 fingers × 3 trials).

### 2.5. Subjective Ratings of the Presence of the Real Exposure

At the end of the second session, participants were asked the following question (in Hungarian): “What do you think, in which of the two sessions did you get exposed to the actual, Real irradiation? At the first (A) session? /At the second (B) session? /No idea?” Six of them correctly identified the session of Real exposure, 9 participants gave a wrong answer and 3 could not tell the answer. When A and B responses in which the subjects expressed their uncertainty (saying e.g. “…but it is only a guess”) were counted as “No idea,” the proportion of right and wrong answers was as follows: 2 right, 8 wrong, 8 no idea. Thus, we concluded that participants really could not determine when the Real LTE exposure was applied.

### 2.6. Perceived Subjective Pain During the Blocks of Thermal Pain Threshold Trials

After each block of experiments, participants were asked to report on a Visual Analogue Scale (VAS) how painful they perceived the stimuli of individual blocks. The scale was a 10 cm long printed line on a sheet with the left endpoint of the “no pain” sensation and the right endpoint being of the “greatest pain” sensed. The participants’ markings were measured from left to right and the subjective pain sensation was expressed in mm.

### 2.7. Statistical Analysis

To explore the effect of LTE exposure on TPT, the average TPT data per group were calculated. Separate rANOVAs were performed in the control block (Pre), exposure blocks (Mid: block A and B) and post exposure blocks (Post: Block C and D). In the control block we checked whether the control blocks of the two sessions are from the same distribution and how the trials evolve within a block: rANOVA exposure (control_Real_, control_Sham_) × trial (1, 2, 3rd). In the case of exposure blocks and post exposure blocks, the thermal pain induction’s side—the contralateral (CL) or ipsilateral (IL) side relative to the exposure side—on TPT was also investigated. For this purpose, four-factor rANOVAs were used. In the case of TPTs during the exposure (Mid), rANOVA constructed of: exposure (Real, Sham) × block (block A, block B) × side (CL, IL) × trial (1, 2, 3rd). For post exposure TPTs, rANOVA was: exposure (Real, Sham) × block (block C, block D) × side (CL, IL) × trial (1, 2, 3rd). If justified, follow-up analyses were performed using additional ANOVAs.

The analysis of subjective pain sensation was carried out similar compared to TPT data. We analyzed the VAS data with rANOVAs as follows: exposure (Real, Sham) × block (Mid blocks—A and B) or (Post blocks—C and D). Additionally, a t-test was performed to compare the subjective pain sensation in the two sessions of the control block and then to compare the control block to the exposure blocks (block A, block B) and to the post exposure blocks (block C, block D).

The alpha level was set on 0.05 and Tukey correction was used for multiple comparisons. Assumption checks of normality and of sphericity (Mauchly’s Test of Sphericity) were conducted. In cases in which sphericity had been violated, a Greenhouse-Geisser correction was used and degrees of freedom are reported with one decimal digit. Partial eta squared (η^2^*_p_*) are reported as effect size. For the post hoc tests, we reported the mean difference (MD).

## 3. Results

### 3.1. Thermal Pain Threshold Before the Radiofrequency Exposure (Pre)

A significant main effect of trial (F_1.2,21.1_ = 11.628, *p* = 0.002, η^2^*_p_* = 0.406) was found, of which post-hoc analysis suggests (trial_1–2_ t_34_ = −2.42, *p* = 0.053, MD = −0.672; trial_2–3_ t_34_ = −2.40, *p* = 0.056, MD = −0.667; trial_1–3_ t_34_ = −4.82, *p* < 0.001, MD = −1.339) that the TPT increases in consecutive trials of the control block. No exposure main effect or interaction were found (F < 0.78, *p* > 0.46 overall—exposure: F_1,17_ = 0.229, *p* = 0.638, η^2^*_p_* = 0.013), that is the trials of the control blocks in the two sessions are considered the same.

### 3.2. Thermal Pain Threshold During Radiofrequency Exposure (Mid)

**Figure 3:**
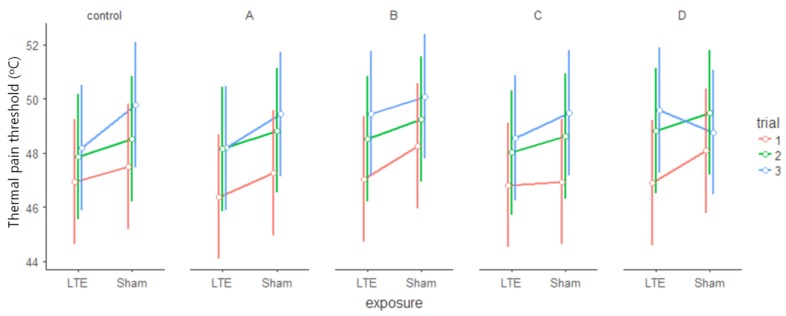
Thermal pain thresholds elevated in trials of the individual blocks. Mean TPTs (°C) of blocks (control, A–D) of trials (1, 2, 3rd) in the Real (LTE) and in the Sham (Sham) condition. Error bars: confidence intervals (95%).

A significant main effect of block (F_1,16_ = 6.480, *p* = 0.022, η^2^*_p_* = 0.288) and significant main effect of trial (F_2, 32_ = 53.348, *p* < 0.001, η^2^*_p_* = 0.769) was found. According to post-hoc test each trial was different from the others (trial_1–2_ t_32_ = −7.13, *p* < 0.001, MD = −1.510; trial_2–3_ t_32_ = −2.90, *p* = 0.018, MD = −0.615, trial_1–3_ t_32_ = −10.04, *p* < 0.001, MD = −2.125), with elevating TPTs (see Figure 3). A marginally significant exposure × side interaction (F_1,16_ = 3.249, *p* = 0.090, η^2^*_p_* = 0.169) was found but post hoc analysis did not indicate that exposure would have differently affected the left or the right side (*p* > 0.35 in all comparisons, the greater MD = −1.009). The marginally significant side × trial interaction (F_2,32_ = 2.494, *p* = 0.098, η^2^*_p_* = 0.135) was due to greater differences between trials on the left (contralateral) side than on the right (ipsilateral) side (the largest difference on the left: left_MDmax_ = −2.31, the largest mean difference on the right: right_MDmax_ = −1.94). However, the related trials of the left and right sides were not different (trial1_left-right_, trial2_left-right_, trial3_left-right_ *p* > 0.60 in all three cases). Other comparisons showed no significant differences (F < 1.67, *p* > 0.21 everywhere—exposure main effect (F_1,16_ = 1.320, *p* = 0.267, η^2^*_p_* = 0.076).

In the case of a simpler three-way rANOVA (exposure (LTE, Sham) × block (Block A, Block B) × trial (1, 2, 3rd)), no significant exposure main effect was found (F_1,17_ = 1.705, *p* = 0.209, η^2^*_p_* = 0.091), only the blocks (F_1,17_ = 5.782, *p* = 0.028, η^2^*_p_* = 0.254) and the trials (F_2,34_ = 58.615, *p* < 0.001, η^2^*_p_* = 0.775) differed in the same way as described above (trial_1–2_ t_34_ = −7.43, *p* < 0.001, MD = −1.489; trial_2–3_ t_34_ = −3.11, *p* = 0.010, MD = −0.622; trial_1−3_ t_34_ = −10.54, *p* < 0.001, MD = −2.111). No interaction of variables was found (F < 1.70, *p* > 0.20, η^2^*_p_* < 0.069 overall).

### 3.3. Thermal Pain Threshold After Radiofrequency Exposure (Post)

**Figure 4:**
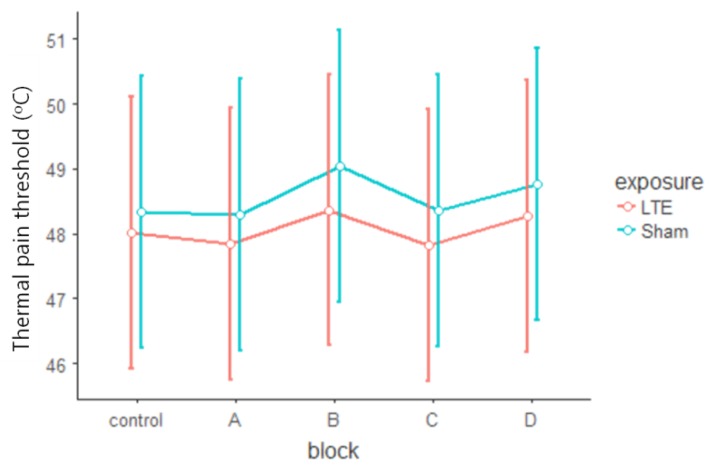
Thermal pain thresholds of the Real and Sham exposure did not differ. Mean TPTs (°C) of blocks (control, A–D) in the Real (LTE) and in the Sham (Sham) condition. Error bars: confidence intervals (95%).

The significant main effect (F_2,28_ = 20.831, *p* < 0.001, η^2^*_p_* = 0.598) also pointed to the increasing tendency of TPTs in the individual blocks (trial_1–2_ t_28_ = −4.88, MD = −1.551, *p* < 0.001, trial_2–3_ t_28_ = −1.22, *p* = 0.449, MD = −0.389; trial_1–3_ t_28_ = −6.10, *p* < 0.001, MD = −1.940). The origin of the significant exposure × block × side × trial interaction (F_2,28_ = 3.433, *p* = 0.046, η^2^*_p_* = 0.197) was investigated with two additional rANOVAs, one for Real and another for Sham exposure. Both in the Real (Real block × side × trial rANOVA) and the Sham exposure (Sham block × side × trial rANOVA) there was a clear difference between the trials (Real: F_2,32_ = 49.532, *p* < 0.001, η^2^*_p_* = 0.756; Sham: F_2,30_ = 8.635, *p* = 0.001, η^2^*_p_* = 0.365). The post-hoc analysis showed that there were greater differences between the trials during Real exposure (Real: trial_1–2_ t_32_ = −7.10, *p* < 0.001, MD = −1.497; trial_2–3_ t_32_ = −2.50, *p* = 0.046, MD = −0.526; trial_1-3_ t_32_ = −9.59, *p* < 0.001, MD = −2.024) than in the Sham (Sham: trial_1-2_ t_30_ = −3.42, *p* < 0.005, MD = −1.614; trial_2–3_ t_30_ = −0.33, *p* = 0.942, MD = −0.155; trial_1–3_ t_30_ = −3.75, *p* = 0.002, MD = −1.769). The marginally significant differences that were not related to exposure (Real: marginal block main effect F_1,16_ = 4.397, *p* = 0.052, η^2^*_p_* = 0.216, Sham: marginal side × trial interaction F_2,30_ = 2.996, *p* = 0.065, η^2^*_p_* = 0.166) were not studied further. No other comparisons indicated that the two groups would differ (Real: F < 1.75, *p* > 0.20, η^2^*_p_* < 0.099 overall, Sham F < 3.00, *p* > 0.19, η^2^*_p_* < 0.108 overall). The blockwise plots of TPTs see on Figure 3 and Figure 4.

### 3.4. Perceived Subjective Pain Based on the Visual Analouge Scale

In the pre-exposure control block, there was no significant exposure (LTE, Sham) effect on subjective pain values (t_17_ = −0.366, *p* = 0.719, Cohen’s d = 0.086).

The analysis of the exposure blocks (block A, block B) revealed a significant block main effect (F_1,17_ = 4.555, *p* = 0.048, η^2^*_p_* = 0.211). From the A block to the B block, the subjective pain sensation increased both in the LTE and in the Sham conditions. Paired *t*-tests showed that none of the exposure blocks (A, B) differed from the control block (t_17_ < −1.66, *p* > 0.11).

Analysis of post exposure blocks (block C, block D) did not show any significant main effects or interactions (F_1,17_ < 1.136, *p* > 0.300, η^2^*_p_* < 0.064). None of the post exposure blocks differed significantly from the control block (t_17_ < 1.26, *p* > 0.224). The findings above suggest that subjective pain was not affected by LTE exposure.

The Bonferroni-corrected paired-sample t-tests indicated that control blocks did not differ between Real and Sham exposure. No difference was obtained between the control block and either of the exposure blocks or post exposure blocks, nor in the LTE, neither in the Sham condition. This finding suggests that, compared to the pre-exposure control, participants did not show altered sensitivity to thermal pain across the two exposure conditions. Table 1 and Table 2 show the descriptive and the *t*-statistics on VAS ratings.

In summary, analysis of TPT data from the current study demonstrates that LTE exposure had no effect on thermal pain threshold either during the 30-min exposure period or in the subsequent 60 min. This is consistent with the subjective pain-related results showing no change of perceived pain due to LTE exposure.

## 4. Discussion

In the present study we investigated the effect of 4G (LTE) MP-like RF exposure on thermal pain threshold in a randomized, double-blind, counterbalanced experiment using the protocol developed in our previous study [25] where we examined the effects of 3G (UMTS) MPs on the same TPT endpoint. In that study, the UMTS EMF exposure was found to strengthen the thermal pain desensitization related to repeated noxious stimulation, as reflected in a change in the rate of increase in TPTs throughout the 3 trials of a six-min stimulation blocks during RF exposure. In the present study, however, with exactly the same methodology and experimental protocols, we failed to show any observable effects of LTE RF exposure on TPT.

A possible reason behind the discrepancies in the results of the two studies can be the different physical parameters of the RF EMF exposure types. The carrier frequency (1945 MHz) of the used UMTS system differed almost 200 MHz from the LTE system (1750 MHz) and therefore has a slightly different penetration depth. More importantly, the nature of their modulation (modulation bandwidth) is also different. The UMTS signal has an amplitude modulation of 1500 Hz, which means that the signal changes its intensity at 1500 Hz. By contrast, the envelope of the LTE signal, due to the superposition of the multiple carrier signals differing in phase and modulation, resembles the CW (continuous wave) RF signal. Current models on the mechanisms behind the MP-like EMF effects emphasize the key role of modulation. This may explain that while there was some mild effect of UMTS exposure, and such an effect could not be demonstrated for LTE exposure.

In our previous study on TPT, a small and short-term effect of UMTS RF exposure was found with the same experimental protocol [25]. The strength of the present study is its comparability with a previously conducted measurement under similar experimental conditions and thereby testing non-thermal effects of distinct types (UMTS, LTE) of RF exposure with precisely characterized exposure parameters. However, our conclusions are limited only to the threshold on thermal pain induced peripherally on the subject’s hand and not at the exposure area of the handheld devices. Although it is reassuring to note that short term (30-min) acute RF-EMF radiation did not cause any alterations in pain perception, it cannot be ruled out that a longer-term (e.g., more than one-hour) real exposure could affect TPT.

Many studies have dealt with the potential effects of the former Global System for Mobile Communications (GSM) technology on self-reported symptoms such as headaches incl. migraine, sleep disorders, forgetfulness and skin irritation [29,30,31,32]. The findings of these studies are not consistent with the increase in adverse symptoms associated with EMF exposure by MPs. Possible effects of UMTS base stations on wellbeing were studied under double blind experiments using base station-like exposure [33] and no correlation was found between exposure levels and mood.

So far, no reports of adverse effects of EMFs on wellbeing and symptoms have been substantiated, except for pain (and potentially tissue damage), which is related to elevated temperature at high exposure levels. According to Walters et al. [14] RF-EMF at sufficiently high levels can cause pain, as exposure to 3-s 12.5 kW/m^2^ of 94 GHz high frequency EMF to the back induced pain threshold (which raised temperature at a heat rate of 3.3 °C per second from 34 °C to 43.9 °C). This finding is similar to those of reported using heat sources other than EMF, where ‘weak to moderate’ pain was reported for smaller temperature elevations (+4 °C) but with a similar rate of temperature elevation (4 °C per s) [34]. However, the latter effect was in the range of millimeter waves and was mainly due to the heat effect of the RF EMF. In our present study, the exposure parameters of RF EMF exposure were set not to cause tissue heating, so we aimed at observing the non-thermal effects of RF EMF on TPT.

This study takes a step forward in assessing the potential effects of 4G technology on pain perception. However, there are some limitations that future studies might aim to address. One issue is that unfortunately the size of our sample (N = 18) is arguably lower than what might be desirable to achieve statistical power to detect potentially small effects. Also, this study is exploratory, that is, we did not have a strong a priori hypothesis that we could use to constrain our analyses, for example, whether and how the exposure would affect TPT during or after exposure. Thus, future confirmatory studies improving their statistical power with more focused hypothesis testing and larger sample sizes could further confirm that 4G (LTE) technology does not affect pain perception. Also, it would have been very interesting to investigate TPT changes not only on the fingers but near the area of realistic MP use (next to the ear), which was not practically feasible with the equipment used in the current study.

In conclusion, the present study demonstrated that 30 min LTE RF exposure emitted by 4G MPs on volunteers’ head did not alter the peripheral TPT and the associated subjective pain sensation on healthy young adults under controlled laboratory conditions. Results are in line with other experimental studies showing that apparent symptoms of pain or sensitization to pain attributed to MP use are usually unrelated to the actual RF exposure but rather closely follows the predispositions of the user towards these devices manifesting as nocebo or placebo effects [1,35,36].

## Figures and Tables

**Table 1:** Means and standard deviations of subjective pain ratings assessed using the Visual Analogue Scale for each experimental block.

Control Blocks			Exposure Blocks				Post Exposure Blocks			
			Block A		Block B		Block C		Block D	
	Sham	LTE	Sham	LTE	Sham	LTE	Sham	LTE	Sham	LTE
mean	45.9	44.4	46.4	45.6	49.8	48.1	48.7	45.5	49.7	47.1
SD	20.6	19.7	21.8	20.5	22.7	19.8	21	21.9	23.8	21.5

**Table 2:** Results of paired samples *t*-tests of subjective pain ratings assessed using the Visual Analogue Scale (VAS) for each experimental block.

Paired Samples *t*-test (Student’s t)		VAS	
		*t* Statistic	*p*
LTE control	Sham control	−0.366	0.719
LTE control	LTE block A	−1.140	0.270
	LTE block B	−1.651	0.117
	LTE block C	-0.420	0.680
	LTE block D	−1.014	0.325
Sham control	Sham block A	−0.195	0.848
	Sham block B	−1.516	0.148
	Sham block C	−1.176	0.256
	Sham block D	−1.258	0.225

Note: Bonferrroni-adjusted alpha level = 0.05 × 9 = 0.006, df = 17.
